# Neuronal Machinery of Sleep Homeostasis in *Drosophila*

**DOI:** 10.1016/j.neuron.2013.12.013

**Published:** 2014-02-19

**Authors:** Jeffrey M. Donlea, Diogo Pimentel, Gero Miesenböck

**Affiliations:** 1 Centre for Neural Circuits and Behaviour, University of Oxford, Tinsley Building, Mansfield Road, Oxford OX1 3SR, UK

## Abstract

Sleep is under homeostatic control, but the mechanisms that sense sleep need and correct sleep deficits remain unknown. Here, we report that sleep-promoting neurons with projections to the dorsal fan-shaped body (FB) form the output arm of *Drosophila*’s sleep homeostat. Homeostatic sleep control requires the Rho-GTPase-activating protein encoded by the *crossveinless-c* (*cv-c*) gene in order to transduce sleep pressure into increased electrical excitability of dorsal FB neurons. *cv-c* mutants exhibit decreased sleep time, diminished sleep rebound, and memory deficits comparable to those after sleep loss. Targeted ablation and rescue of Cv-c in sleep-control neurons of the dorsal FB impair and restore, respectively, normal sleep patterns. Sleep deprivation increases the excitability of dorsal FB neurons, but this homeostatic adjustment is disrupted in short-sleeping *cv-c* mutants. Sleep pressure thus shifts the input-output function of sleep-promoting neurons toward heightened activity by modulating ion channel function in a mechanism dependent on Cv-c.

## Highlights

• Neurons in the dorsal fan-shaped body convey the output of the sleep homeostat
• Sleep need increases the electrical excitability of these sleep-control neurons
• Sleep homeostasis requires the Rho-GAP encoded by the *crossveinless-c* gene
• Cv-c modulates ion channel function in sleep-control neurons

## Teaser

Donlea et al. discover mutant flies with a broken sleep homeostat: after an all-nighter, these flies are unable to correct the accumulated sleep deficit. The mutation affects a Rho-GTPase-activating protein that regulates the electrical excitability of sleep-promoting neurons.

## Introduction

Sleep and wakefulness are regulated by separate but interacting circadian and homeostatic systems (Borbély, 1982). The circadian system allows animals to anticipate regularly recurring external changes caused by the Earth’s rotation, whereas the homeostatic system senses still ill-defined internal changes thought to accumulate during waking and enables their reset by vital, but also ill-defined, functions of sleep.

The discovery of the molecular and cellular mechanisms underpinning circadian sleep control is one of the triumphs of behavioral genetics. After the isolation of *period*, a *Drosophila* mutant with altered circadian timekeeping (Konopka and Benzer, 1971), much has been learned about the composition and function of the circadian clock. We now understand that the molecular clock consists of negative feedback loops in which proteins encoded by clock genes (Bargiello et al., 1984; Reddy et al., 1984; Reick et al., 2001; Sehgal et al., 1994; Vitaterna et al., 1994) inhibit their own transcription, resulting in oscillatory gene expression (Allada et al., 1998; Darlington et al., 1998; Rutila et al., 1998). Transcriptional clocks operating throughout the body are synchronized by pacemaker neurons in the brain (Welsh et al., 1995; Yoo et al., 2004). These neurons and the signals they emit in order to entrain subordinate oscillators have been identified in several species. For examples, the pigment-dispersing factor (PDF)-expressing lateral neurons in *Drosophila* impose their rhythm through the timed release of the neuropeptide PDF (Ewer et al., 1992; Renn et al., 1999; Siwicki et al., 1988); clock neurons in the suprachiasmatic nucleus of mammals (Lehman et al., 1987; Ralph et al., 1990) communicate with peripheral oscillators by secreting a variety of peptides, including transforming growth factor α (Kramer et al., 2001), prokineticin 2 (Cheng et al., 2002), and cardiotrophin-like cytokine (Kraves and Weitz, 2006). Many pacemaker neurons display daily variations in electrical activity that are influenced by, and influence, the molecular clock (Cao and Nitabach, 2008; Nitabach et al., 2002; Welsh et al., 1995).

By comparison, very little is known about the neural mechanisms of sleep homeostasis. Although genetic analyses have begun to identify loci that affect homeostatic sleep control in flies (Bushey et al., 2009; Ishimoto and Kitamoto, 2010; Koh et al., 2008; Shaw et al., 2002), mice (Franken et al., 2001; Kapfhamer et al., 2002), and humans (Viola et al., 2007), these analyses have not yet unearthed a Rosetta Stone akin to *period*. Several studies have implicated clock components also in sleep homeostasis (Naylor et al., 2000; Shaw et al., 2002; Viola et al., 2007), but it remains unclear whether these genes influence circadian and homeostatic processes independently or through shared pathways. Some of the genes linked specifically to sleep homeostasis, such as molecular chaperones (Shaw et al., 2002), components of steroid signaling systems (Ishimoto and Kitamoto, 2010), or unidentified quantitative trait loci (Franken et al., 2001), lack unique or well-defined roles in neuronal physiology that could point to particular regulatory mechanisms. Still others, such as modulators of potassium currents (Koh et al., 2008), Ca^2+^-regulated synaptic vesicle release (Kapfhamer et al., 2002), or synaptogenesis (Bushey et al., 2009), hint that sleep homeostasis—like many other forms of information processing in the nervous system—might involve changes in electrical activity or synaptic communication. However, there has been no indication of the specific nature of these changes, the sites where they occur, or their mechanistic relationship to sleep control.

The identification of molecular and cellular machinery of sleep homeostasis is a prerequisite for understanding how neural circuits monitor an animal’s sleep history, compare the resulting sleep balance to a set point, and correct sleep deficits. A variety of neural structures in mammals have been implicated in the regulation of sleep, but these nuclei all consist of heterogeneous cell groups whose functions have been difficult to resolve (for reviews, see Brown et al., 2012, and Saper et al., 2010). In light of this complexity, the recognition that sleep loss in *Drosophila* causes behavioral and cognitive deficits comparable to those in mammals (Bushey et al., 2007; Li et al., 2009b; Seugnet et al., 2008; Shaw et al., 2002) has spurred attempts to dissect neural mechanisms of sleep regulation in the fly. Recent studies have pinpointed genetically circumscribed neuronal populations that influence sleep, including cells among the lateral neurons of the circadian circuitry (Parisky et al., 2008; Sheeba et al., 2008), the mushroom body (Joiner et al., 2006; Pitman et al., 2006), the pars intercerebralis (Crocker et al., 2010; Foltenyi et al., 2007), and elements of neuromodulatory systems (Andretic et al., 2005; Crocker et al., 2010; Kume et al., 2005; Liu et al., 2012; Ueno et al., 2012). Dopaminergic arousal signals (Andretic et al., 2005; Kume et al., 2005) modulate the activity of a cluster of neurons with projections to the dorsal fan-shaped body (FB) (Liu et al., 2012; Ueno et al., 2012) whose artificial activation induces sleep on demand (Donlea et al., 2011). Because dorsal FB neurons also mediate sensitivity to general anesthetics (Kottler et al., 2013), they are reminiscent in at least two respects of sleep-active neurons in the hypothalamic ventrolateral preoptic nuclei of mammals whose activity is similarly correlated with sleep (Sherin et al., 1996) and stimulated by hypnotic anesthetics (Lu et al., 2008; Moore et al., 2012; Nelson et al., 2002). Here, we show that the sleep-control neurons of the dorsal FB form the output arm of the fly’s sleep homeostat and delineate a mechanism that regulates their activity in response to sleep need.

## Results

### Mutations of the Rho-GTPase-Activating Protein Cv-c Cause Sleep Loss

**Figure 1:**
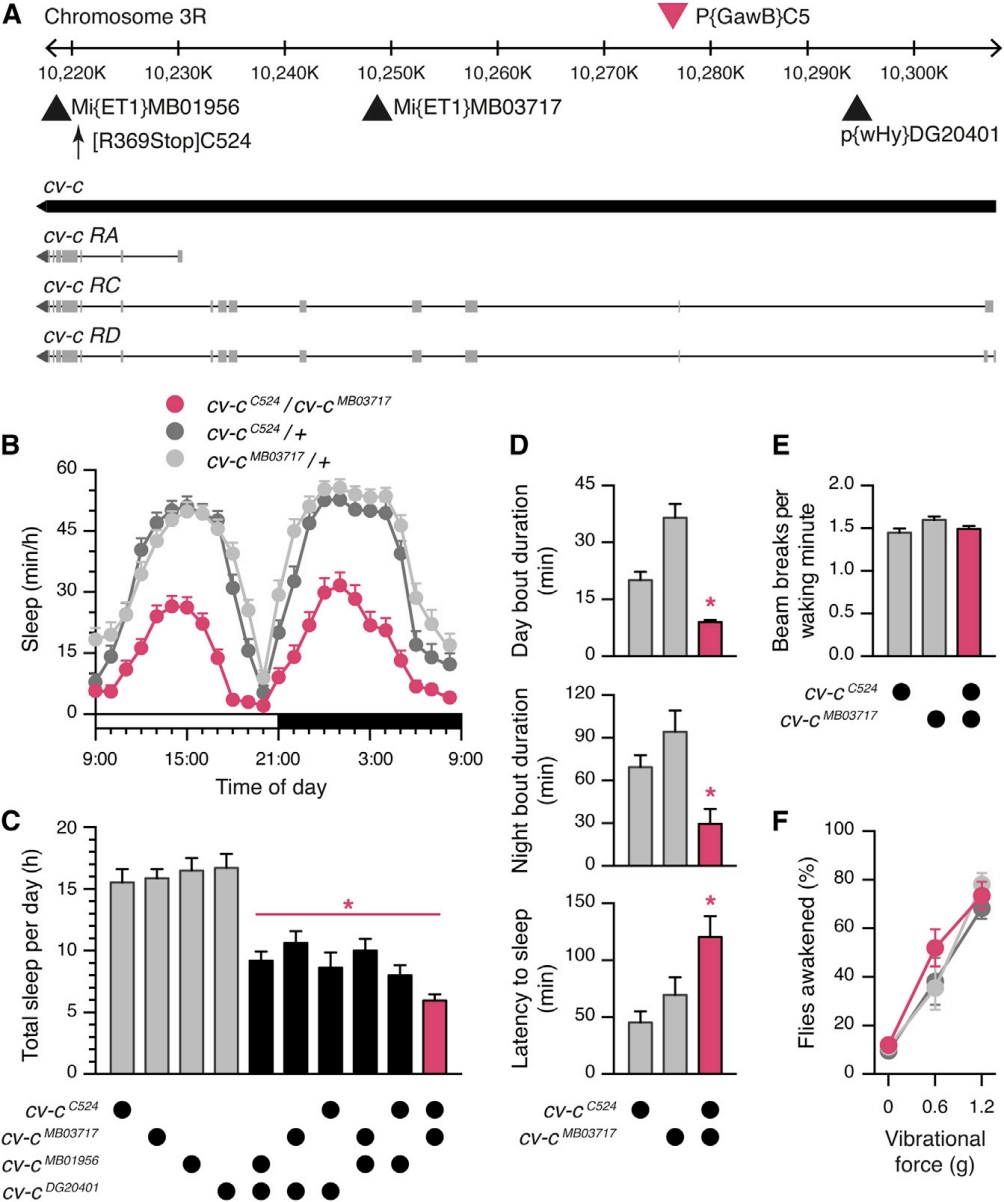
Mutations in *cv-c* Reduce Sleep (A) The P element insertion site in the *C5-GAL4* line (red triangle), as determined by splinkerette PCR, maps to an intronic region of the *cv-c* locus. The sites affected by mutation in the four *cv-c* alleles used in this study (black triangles for transposon insertions, vertical arrow for nonsense mutation) are indicated with respect to the exon-intron structure of three annotated *cv-c* transcripts. (B) *cv-c*^*C524*^*/cv-c*^*MB03717*^ mutant flies sleep less than *cv-c*^*C524*^*/+* or *cv-c*^*MB03717*^*/+* heterozygous controls during a 24-hr day (mean ± SEM, n = 48 flies per group). Repeated-measures ANOVA detected a significant time × genotype interaction (F_[46, 3384]_ = 4.22, p < 0.0001). One-way ANOVA detected a significant genotype effect on total sleep time per day (F_[2,141]_ = 70.03, p < 0.0001). (C) Flies carrying heteroallelic combinations of four independent *cv-c* mutant alleles (*cv-c*^*DG20401*^, *cv-c*^*MB01956*^, *cv-c*^*MB03717*^, and *cv-c*^*C524*^; heteroallelic genotypes shown in black or red) exhibit reduced sleep time in comparison to heterozygous controls (gray; mean ± SEM, n = 15–48 flies per group). One-way ANOVA detected a significant genotype effect (F_[9,180]_ = 24.97, p < 0.0001). Asterisks denote significant differences from heterozygous controls in pairwise post hoc comparisons. (D) *cv-c*^*C524*^*/cv-c*^*MB03717*^ mutants (red) exhibit reduced daytime sleep bout duration (top), reduced nighttime sleep bout duration (center), and prolonged latency to sleep onset after the fall of darkness (bottom) relative to *cv-c*^*C524*^*/+* or *cv-c*^*MB03717*^*/+* controls (gray; mean ± SEM, n = 47–48 flies per group). One-way ANOVA detected significant genotype effects for day-time sleep bout duration (F_[2,140]_ = 29.64, p < 0.0001), nighttime sleep bout duration (F_[2,141]_ = 8.00, p = 0.0005), and latency to sleep onset (F_[2,141]_ = 6.49, p = 0.002). Asterisks denote significant differences from heterozygous controls in pairwise post hoc comparisons. (E) *cv-c*^*C524*^*/cv-c*^*MB03717*^ mutants (red) show no evidence of hyperactivity, as measured by locomotor counts per waking minute, relative to *cv*-*c*^*C524*^*/*+ and *cv-c*^*MB03717*^/+ controls (gray; mean ± SEM, n = 48 flies per group). One-way ANOVA failed to detect a significant genotype effect (F_[2,141]_ = 2.89, p = 0.06). (F) *cv-c*^*C524*^*/cv-c*^*MB03717*^ mutants (red) show no evidence of altered arousal thresholds during sleep relative to *cv-c*^*C524*^*/+* or *cv-c*^*MB03717*^*/+* controls (gray; mean ± SEM, n = 5–6 trials per group). Two-way ANOVA detected a significant effect of stimulation intensity (F_[2,42]_ = 76.79, p < 0.0001) but failed to detect a significant genotype effect (F_[2,42]_ = 1.057, p = 0.36). See also Figure S1.

To identify molecular machinery that might regulate sleep from within the dorsal FB, we mapped the genomic insertion sites of P elements in *C5-GAL4* (Yang et al., 1995), *104y-GAL4* (Rodan et al., 2002; Sakai and Kitamoto, 2006), and *C205-GAL4* (Martin et al., 1999), which are all enhancer trap lines that can be used to modulate sleep by manipulating dorsal FB activity (Donlea et al., 2011; Kottler et al., 2013; Liu et al., 2012; Ueno et al., 2012). Whereas the transposon insertion sites in *104y-GAL4* and *C205-GAL4* lie in intergenic regions (Figures S1A and S1B available online), the P element in *C5-GAL4* is located within an intron of the *crossveinless-c* (*cv-c*) gene (Figure 1A), which encodes a Rho-GTPase-activating protein (Rho-GAP) (Denholm et al., 2005).

To test for a potential role of Cv-c in sleep regulation, we observed the sleep patterns of flies carrying mutant *cv-c* alleles. Given that homozygotes for several severe *cv-c* alleles do not survive to adulthood, we measured sleep in transheterozygous allelic combinations. As shown in Figures 1B and 1C, these transheterozygotes slept much less than heterozygous controls did, exhibited reduced sleep bout duration during the day and night (Figure 1D, top and center), and displayed prolonged latency to sleep onset at the beginning of the night (Figure 1D, bottom). Importantly, *cv-c*^*C524*^*/cv-c*^*MB03717*^ mutants showed no change in the intensity of waking locomotor activity (Figure 1E) or the levels of arousal thresholds during sleep when compared to heterozygous controls (Figure 1F). This suggests that the mutant’s decreased sleep time is not a consequence of hyperactivity due to heightened arousal.

We further verified that the insomnia of mutants was the result of molecular lesions at the *cv-c* locus by examining sleep patterns in heteroallelic combinations of four independently generated mutant alleles. All of the tested allelic combinations exhibited decreases in total sleep time relative to heterozygous controls (Figure 1C). Altogether, these results demonstrate that mutations in *cv-c* interfere with the initiation and/or maintenance of sleep.

### Cv-c Is Required for Homeostatic Sleep Control

**Figure 2:**
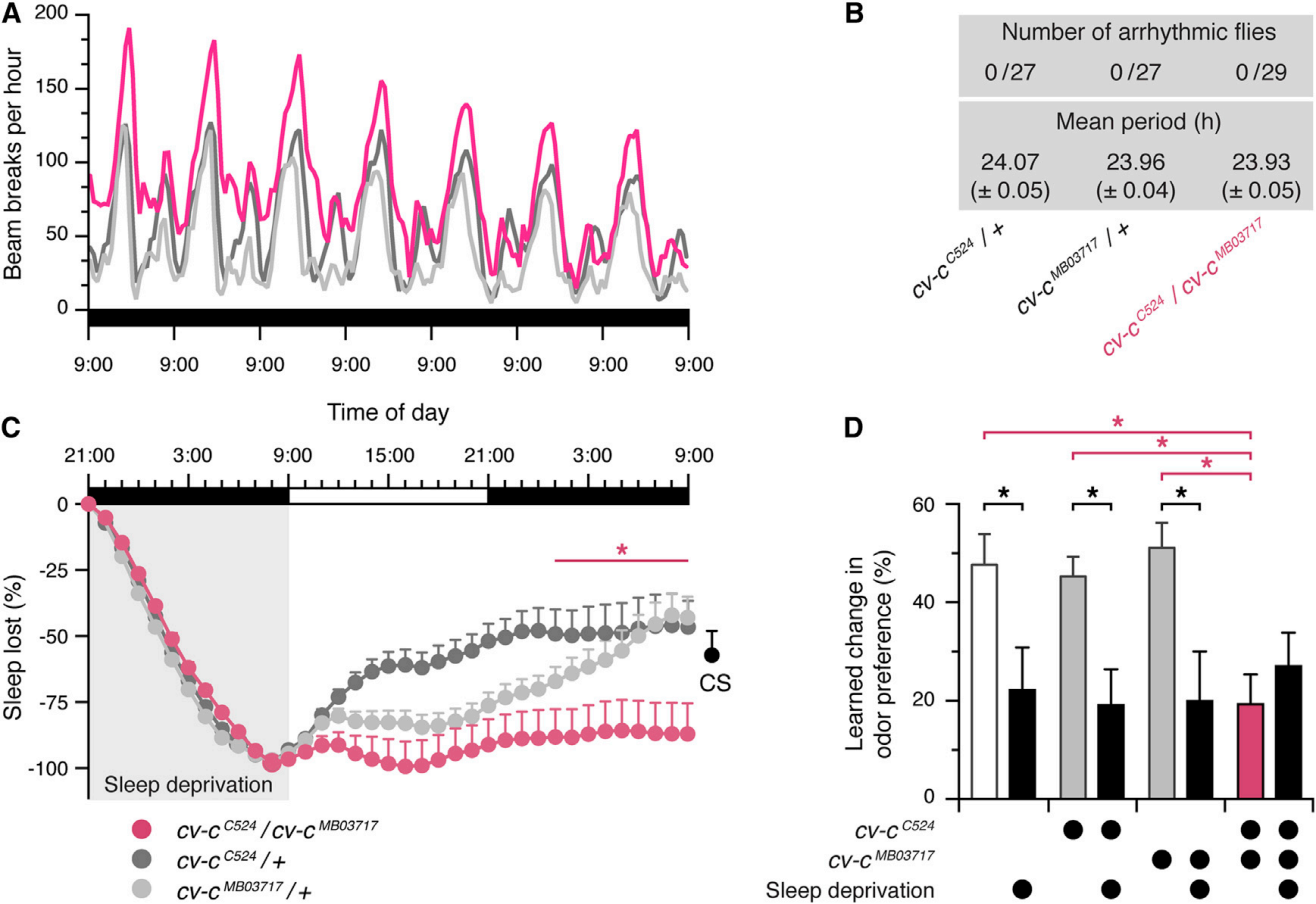
Mutations in *cv-c* Impair Homeostatic Sleep Regulation (A and B) *cv-c*^*C524*^*/cv-c*^*MB03717*^ mutants show no change in circadian locomotor rhythmicity during 7 days in constant darkness. One-way ANOVA failed to detect a significant genotype effect on circadian period (F_[2,78]_ = 2.618, p = 0.08). (C) After overnight sleep deprivation for 12 hr, *cv-c*^*C524*^*/cv-c*^*MB03717*^ mutants regain a significantly lower percentage of their lost sleep than heterozygous controls (mean ± SEM, n = 67–85 flies per group). Two-way repeated-measures ANOVA of sleep percentage regained detected a significant genotype × time interaction (F_[70,7875]_ = 5.68, p < 0.0001). Asterisks denote time points at which sleep percentage regained differs significantly between *cv-c*^*C524*^*/cv-c*^*MB03717*^ mutants and both heterozygous controls in pairwise post hoc comparisons. A single data point on the right (black) indicates the amount of sleep regained by Canton-S (CS) flies at the end of a comparable experiment (mean ± SEM, n = 242 flies). (D) Short-term memory is impaired in sleep-deprived CS flies (black, left) in comparison to rested siblings (white; mean ± SEM, n = 29–30 flies per group; two-tailed Student’s t test, p = 0.02). Rested *cv-c*^*C524*^*/cv-c*^*MB03717*^ mutants (red, right) exhibit similarly reduced short-term memory in comparison to heterozygous controls (gray, right). Overnight sleep deprivation decreases short-term memory in heterozygous controls (compare gray and black columns) but not in *cv-c*^*C524*^*/cv-c*^*MB03717*^ mutants (compare red and black columns) (mean ± SEM, n = 37–58 flies per group). Two-way ANOVA detected a significant genotype × sleep history interaction (F_[2,273]_ = 5.35, p = 0.0052). Asterisks denote significant differences in pairwise post hoc comparisons. See also Figure S2.

To distinguish between possible roles of *cv-c* in circadian and homeostatic sleep regulation, we first tested whether the sleep phenotypes of *cv-c* mutants could be attributed to disruption of the circadian clock. *cv-c*^*C524*^*/cv-c*^*MB03717*^ mutants and heterozygous controls were entrained to a 12 hr light/12 hr dark cycle, and their free-running locomotor rhythms were subsequently analyzed in constant darkness. Over the course of 7 days in darkness, controls and mutants retained robust circadian rhythmicity (Figure 2A). All genotypes exhibited similar mean circadian periods, as measured by χ^2^ periodogram (Figures 2A and 2B), indicating that *cv-c* mutations cause sleep disruptions through pathways that are independent of the circadian clock.

To examine whether the insomnia of *cv-c* mutants might be associated with impaired homeostatic regulation, we mechanically deprived flies of sleep for 12 hr overnight and measured the amount of sleep that was regained over the following 24 hr. *cv-c*^*C524*^*/cv-c*^*MB03717*^ mutants made up for a significantly lower percentage of their lost sleep than either *cv-c*^*C524*^*/*+ or *cv-c*^*MB03717*^/+ controls (Figures 2C and S2A). Although these data demonstrate that the homeostatic response to sleep deprivation is abrogated by a loss of Cv-c function, they do not distinguish between an inability to compensate for sleep loss and an overall reduced sleep requirement of mutants. To differentiate between these possibilities, we measured the ability of flies to form associative short-term memories. Sleep deprivation impairs memory formation in a variety of species, including humans (Stickgold et al., 2001), mice (Florian et al., 2011), and *Drosophila* (Bushey et al., 2007; Li et al., 2009b; Seugnet et al., 2008). As expected, aversive olfactory memory (Claridge-Chang et al., 2009) of individual wild-type (WT) Canton-S (CS) flies was also sensitive to sleep loss: after a night of ad libitum sleep or sleep deprivation, short-term memory was deficient in members of the sleep-deprived group in comparison to their rested siblings (Figures 2D and S2B). Short-sleeping *cv-c*^*C524*^*/cv-c*^*MB03717*^ mutants exhibited memory deficits of the same magnitude as mechanically sleep-deprived flies; these deficits were not exacerbated by further sleep deprivation (Figure 2D). All performance deficits were central in origin, given that neither the flies’ untrained responses to odorants (Figure S2C) nor their sensitivity to electric shock (Figure S2D) varied with sleep history or genotype. These data, along with cell-specific *cv-c* rescue and ablation experiments reported below (see Figure 4), therefore support the hypothesis that flies mutant for *cv-c* have a defect in homeostatic sleep regulation and, as a result, are impaired in the formation of new memories.

### Cv-c Functions in the Dorsal FB to Regulate Sleep

**Figure 3:**
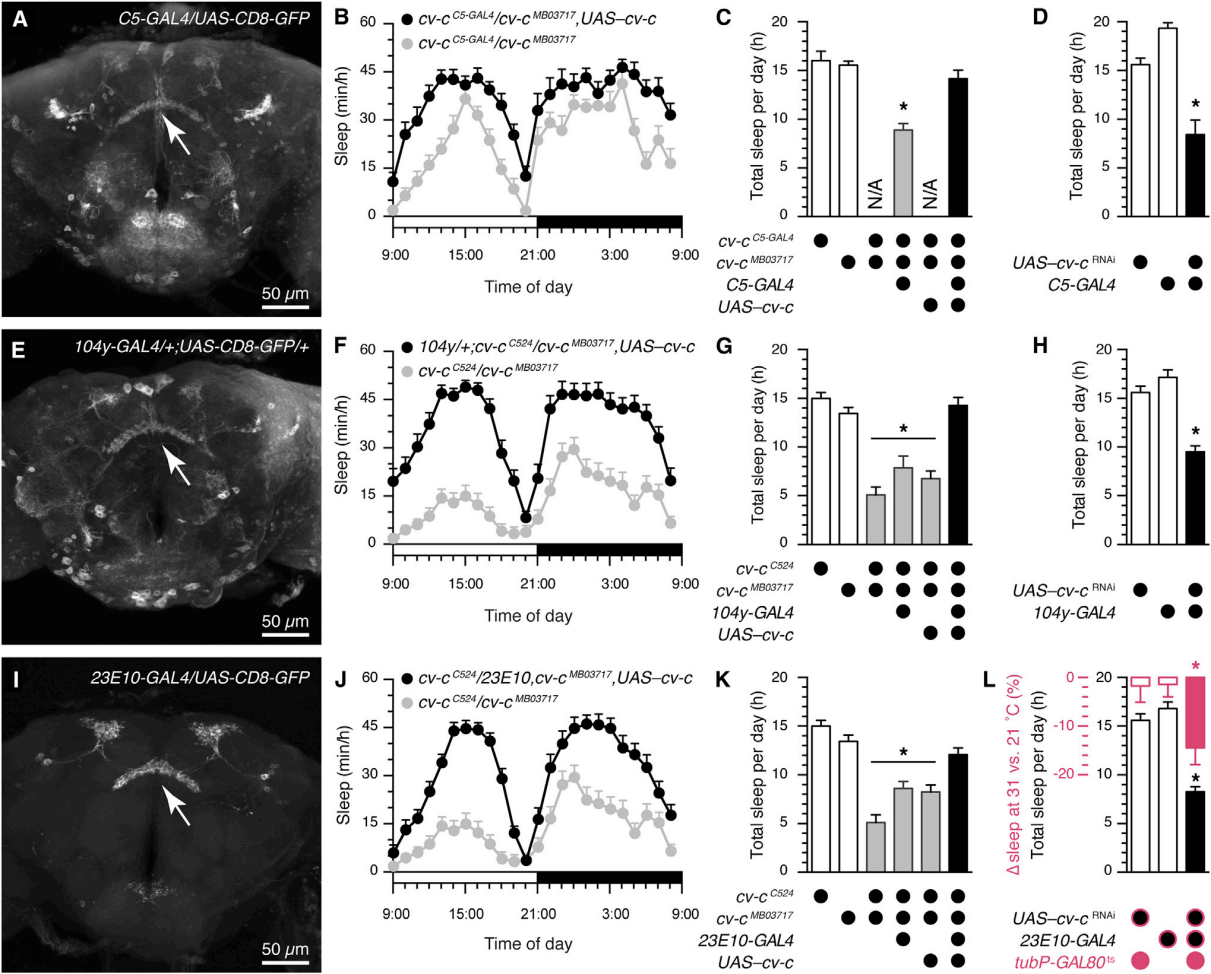
Cv-c Functions in the Dorsal Fan-Shaped Body to Regulate Sleep (A) *C5-GAL4* drives transgene expression in the dorsal FB (arrow). (B) *cv-c*^*C5-GAL4*^*/cv-c*^*MB03717*^ mutants (gray) sleep less than a *cv-c*^*C5-GAL4*^*/cv-c*^*MB03717*^*, UAS–cv-c* rescue strain during a 24-hr day (mean ± SEM, n = 16 flies per group). Two-way repeated-measures ANOVA detected a significant genotype × time interaction (F_[23,690]_ = 1.86, p = 0.0087). (C) Total sleep time is decreased in *cv-c*^*C5-GAL4*^*/cv-c*^*MB03717*^ mutants (gray) in comparison to heterozygous controls (white) and restored to control levels in a *cv-c*^*C5-GAL4*^*/cv-c*^*MB03717*^*, UAS–cv-c* rescue strain (black) (mean ± SEM, n = 16–32 flies per group). One-way ANOVA detected a significant genotype effect (F_[3,75]_ = 20.61, p < 0.0001). The asterisk denotes a significant difference from both heterozygous controls in pairwise post hoc comparisons. (D) Expression of *cv-c*^RNAi^ under the control of *C5-GAL4* (black) reduces sleep compared to parental controls (white; mean ± SEM, n = 14–44 flies per group). One-way ANOVA detected a significant genotype effect (F_[2,71]_ = 24.87, p < 0.0001). The asterisk denotes a significant difference from parental controls in pairwise post hoc comparisons. (E) *104y-GAL4* drives transgene expression in the dorsal FB (arrow). (F) Rescue of *cv-c* under the control of *104y-GAL4* (black) increases sleep in an otherwise mutant *cv-c*^*C524*^*/cv-c*^*MB03717*^ background (gray; mean ± SEM, n = 26–30 flies per group). Two-way repeated measures ANOVA detected a significant genotype × time interaction (F_[23,1242]_ = 4.60, p < 0.0001). (G) Rescue of *cv-c* under the control of *104y-GAL4* (black) increases sleep time from mutant (gray) to control levels (white; mean ± SEM, n = 23–48 flies per group). One-way ANOVA detected a significant genotype effect (F_[5,192]_ = 31.15, p < 0.0001). Asterisks denote significant differences from heterozygous controls in pairwise post hoc comparisons. (H) Expression of *cv-c*^RNAi^ under the control of *104y-GAL4* (black) reduces sleep in comparison to parental controls (white; mean ± SEM, n = 16–44 flies per group). One-way ANOVA detected a significant genotype effect (F_[2,73]_ = 21.09, p < 0.0001). The asterisk denotes a significant difference from both parental controls in pairwise post hoc comparisons. (I) *23E10-GAL4* drives transgene expression exclusively in the dorsal FB (arrow). (J) Rescue of *cv-c* under the control of *23E10-GAL4* (black) increases sleep in an otherwise mutant *cv-c*^*C524*^*/cv-c*^*MB03717*^ background (gray; mean ± SEM, n = 30–31 flies per group). Two-way repeated-measures ANOVA detected a significant genotype × time interaction (F_[23,1357]_ = 6.89, p < 0.0001). (K) Rescue of *cv-c* under the control of *23E10-GAL4* (black) increases sleep time from mutant (gray) to control levels (white; mean ± SEM, n = 30–48 flies per group). One-way ANOVA detected a significant genotype effect (F_[5,212]_ = 29.52, p < 0.0001). Asterisks denote significant differences from heterozygous controls in pairwise post hoc comparisons. (L) Expression of *cv-c*^RNAi^ under the control of *23E10-GAL4* (black) reduces sleep in comparison to parental controls (white; mean ± SEM, n = 22–44 flies per group). One-way ANOVA detected a significant genotype effect (F_[2,94]_ = 38.17, p < 0.0001). Thermally induced expression of *cv-c*^RNAi^ in *UAS-cv-c*^RNAi^*/+; 23E10/tub-GAL80*^ts^ flies at 31°C reduces sleep in comparison to siblings maintained at 21°C (solid red bar); temperature shifts have no effect on parental controls (open red bars; mean ± SEM, n = 43–50 flies per group). One-way ANOVA detected a significant genotype effect (F_[2,138]_ = 5.06, p = 0.0076). Asterisks denote significant differences from both parental controls in pairwise post hoc comparisons.

Because *cv-c* mutations disrupt the sleep homeostat, and because an insertion in the *cv-c* gene drives transgene expression in sleep-promoting neurons of the dorsal FB (among other sites), the dorsal FB neurons themselves might be the site of Cv-c action in sleep control. To test this notion, we restored WT *cv-c* expression in the dorsal FB of otherwise mutant flies. The *cv-c*^*C5-GAL4*^ line, which drives expression in the dorsal FB (Figure 3A), failed to complement *cv-c*^*MB03717*^ (Figures 3B and 3C, gray columns), indicating that the transposon insertion in *C5-GAL4* itself interferes with the function of the *cv-c* gene (Figure 1A). When *cv-c*^*C5-GAL4*^ was used to drive the expression of *UAS*–*cv-c* (Denholm et al., 2005) in the dorsal FB of otherwise mutant *cv-c*^*C5-GAL4*^/*cv-c*^*MB03717*^ flies, sleep returned to WT levels (Figures 3B and 3C, black columns). The spatially restricted RNAi-mediated knockdown of *cv-c* expression in the dorsal FB had the converse effect: driving *UAS*–*cv-c*^RNAi^ (Billuart et al., 2001) with *C5-GAL4* significantly reduced sleep time relative to parental controls (Figure 3D, black column).

Two additional *GAL4* lines that label neurons projecting to the same layer of the FB were used to confirm that *cv-c* acts in the dorsal FB to regulate sleep. The line *104y-GAL4* (Rodan et al., 2002; Sakai and Kitamoto, 2006) labels dorsal FB neurons and a limited number of neurons outside the FB (Figure 3E). Expression of *cv-c* under *104y-GAL4* control in a *cv-c*^*C524*^*/cv-c*^*MB03717*^ mutant background restored WT sleep patterns (Figures 3F and 3G, black columns), whereas the expression of *UAS*–*cv-c*^RNAi^ under *104y-GAL4* control in WT flies significantly decreased sleep time in comparison to the parental strains (Figure 3H, black column).

The line *23E10-GAL4* shows a particularly high degree of specificity for dorsal FB neurons (Jenett et al., 2012), with little or no transgene expression detectable elsewhere (Figure 3I). Localized rescue of *cv-c* in a *cv-c*^*C524*^*/cv-c*^*MB03717*^ background with *23E10-GAL4* restored WT sleep levels (Figures 3J and 3K, black columns); expression of *cv-c*^RNAi^ under *23E10-GAL4* control decreased sleep relative to parental controls (Figure 3L, black column). Thus, the function of Cv-c within dorsal FB neurons is necessary and sufficient for the proper regulation of baseline sleep.

To distinguish an ongoing from a purely developmental role of Cv-c in the dorsal FB, we used a temperature-sensitive repressor of GAL4, GAL80^ts^ (McGuire et al., 2003), to prevent the expression of *cv-c*^RNAi^ prior to adulthood. RNAi was induced by shifting adult *UAS-cv-c*^RNAi^/+;*23E10*/*tub-GAL80*^ts^ flies from a permissive (21°C) to a restrictive (31°C) temperature, and sleep was quantified over the following 2 days. Although inducible RNAi-mediated knockdown of *cv-c* is expected to deplete only part of the pre-existing Cv-c protein pool (at most, the fraction undergoing natural turnover during the 2-day analysis window), experimental flies housed at 31°C lost a significant amount of daily sleep relative to siblings remaining at 21°C (Figure 3L, solid red bar). Temperature shifts had no effect on total sleep time in parental controls (Figure 3L, open red bars).

**Figure 4:**
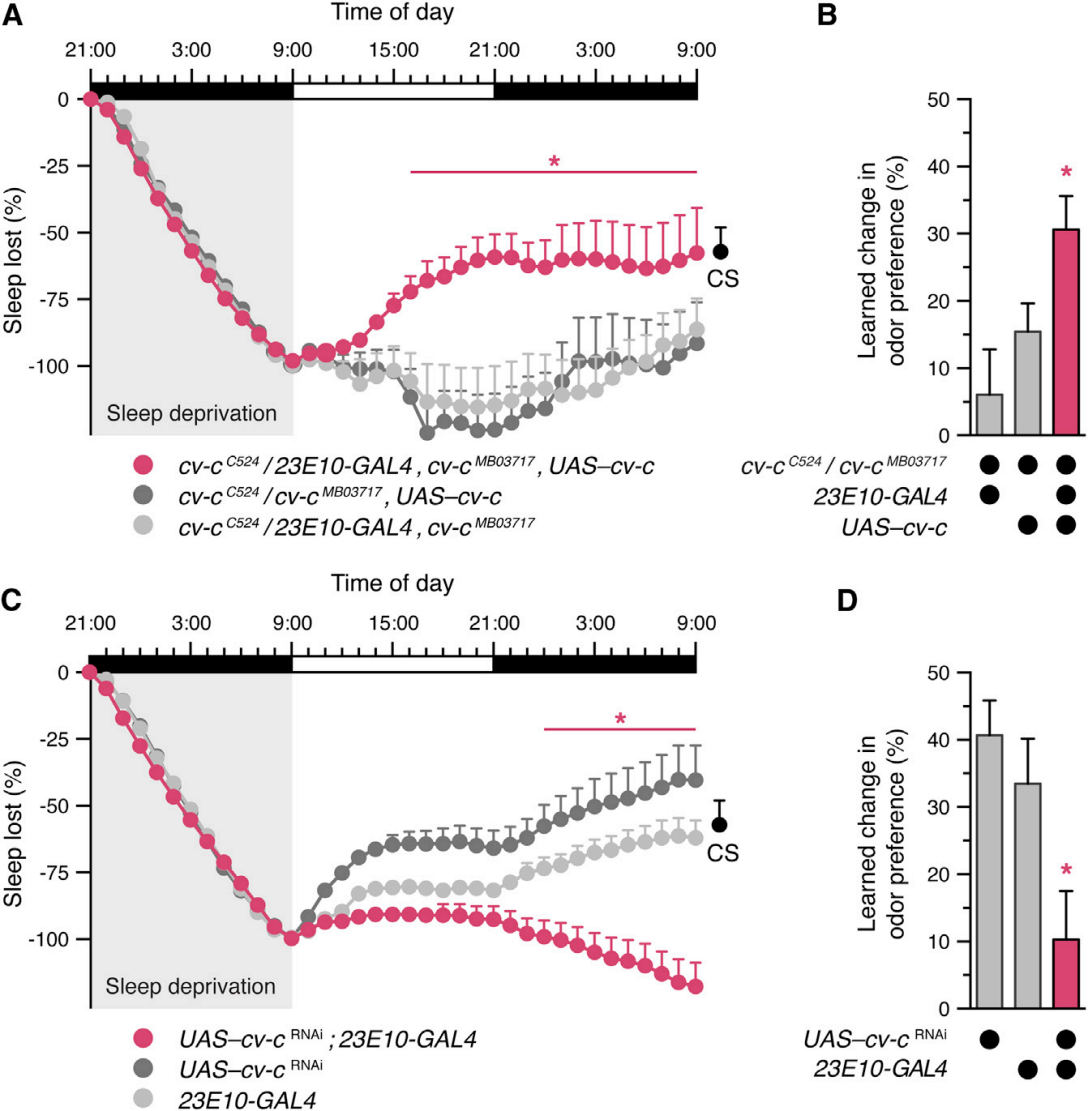
Deficits in Homeostatic Sleep Control and Memory Reflect the Role of Cv-c in the Dorsal Fan-Shaped Body (A) Rescue of *cv-c* under the control of *23E10-GAL4* increases sleep rebound after 12 hr of sleep deprivation in an otherwise mutant background (mean ± SEM, n = 52–61 flies per group). Two-way repeated-measures ANOVA detected a significant genotype × time interaction (F_[72, 6084]_ = 3.84, p < 0.0001). Asterisks denote time points at which sleep percentage regained differs significantly between experimental flies and both parental controls in pairwise post hoc comparisons. A single data point on the right (black) indicates the amount of sleep regained by CS flies at the end of a comparable experiment (mean ± SEM, n = 242 flies). (B) Rescue of *cv-c* under the control of *23E10-GAL4* restores short-term memory in an otherwise mutant background (mean ± SEM, n = 35–91 flies per group). One-way ANOVA detected a significant genotype effect (F_[2,192]_ = 5.01, p = 0.0076). The asterisk denotes a significant difference from both parental controls in pairwise post hoc comparisons. (C) Expression of *cv-c*^RNAi^ under the control of *23E10-GAL4* impairs sleep rebound after overnight sleep deprivation in comparison to parental controls (mean ± SEM, n = 27–110 flies per group). Two-way repeated measures ANOVA detected a significant genotype × time interaction (F_[72, 6804]_ = 6.90, p < 0.0001). Asterisks denote time points at which sleep percentage regained differs significantly between experimental flies and both parental controls in pairwise post hoc comparisons. A single data point on the right (black) indicates the amount of sleep regained by CS flies at the end of a comparable experiment (mean ± SEM, n = 242 flies). (D) Expression of *cv-c*^RNAi^ under the control of *23E10-GAL4* impairs short-term memory in comparison to parental controls (mean ± SEM, n = 40 flies per group). One-way ANOVA detected a significant genotype effect (F_[2,117]_ = 6.13, p = 0.0029). The asterisk denotes a significant difference from both parental controls in pairwise post hoc comparisons. See also Figure S3.

Spatially restricted rescue of *cv-c* expression under the control of *23E10-GAL4* in an otherwise mutant background restored sleep rebound after a night of sleep deprivation (Figures 4A and S3A) and corrected the memory deficit associated with sleep loss (Figures 4B, S3B, and S3C). Conversely, localized ablation of Cv-c in dorsal FB neurons, using *23E10-GAL4* to express *UAS*–*cv-c*^RNAi^, impaired the homeostatic response to sleep loss (Figures 4C and S3D) and caused short-term memory deficits (Figures 4D, S3E, and S3F). These experiments provide direct evidence that Cv-c exerts its role in sleep homeostasis within dorsal FB neurons and that the memory deficits of *cv-c* mutants are secondary to homeostatic sleep dysregulation.

### Cv-c Regulates the Electrical Properties of Dorsal FB Neurons

**Figure 5:**
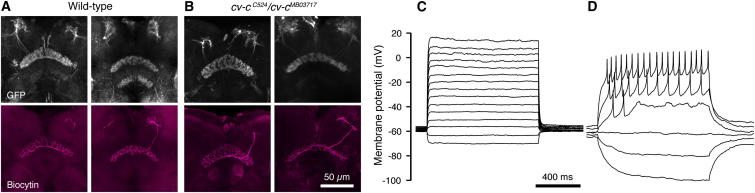
Sleep-Control Neurons Are Morphologically Homogeneous but Electrophysiologically Diverse (A and B) Example morphologies of dorsal FB neurons expressing *UAS–mCD8-GFP* under *104y-GAL4* control in WT (A) or *cv-c*^*C524*^*/cv-c*^*MB03717*^ mutant (B) flies. Top panels show the innervation of the dorsal FB by the genetically labeled neuronal population; the corresponding bottom panels depict individual biocytin-filled neurons in the same individuals. The axons of the *104y-GAL4*-positive neurons span the entire width of the dorsal stratum of the FB (100% ± 0.006% overlap, n = 6 cells). (C and D) Examples of membrane potential changes evoked in dorsal FB neurons by current steps.

Artificial activation of dorsal FB neurons induces sleep (Donlea et al., 2011; Ueno et al., 2012), whereas silencing of dorsal FB neurons, much like disruption of *cv-c* within the same cells (Figure 3), decreases sleep (Kottler et al., 2013; Liu et al., 2012). Cv-c might therefore regulate sleep by modulating the intrinsic electrophysiological properties of dorsal FB neurons. To test this hypothesis, we used targeted whole-cell recordings in *104y-GAL4*;*UAS-CD8-GFP* flies to characterize the spiking patterns and membrane properties of dorsal FB neurons. Dye fills of the recorded neurons showed that each cell innervates the entire width of the 104y-positive stratum in the FB (Figure 5A). Along with a previous study that genetically labeled single cells in this population (Li et al., 2009a), these data indicate that dorsal FB neurons comprise a structurally homogeneous cell group. Mutating *cv-c* caused neither changes in the innervation pattern of the dorsal FB nor conspicuous morphological abnormalities of individual dye-filled cells (Figure 5B).

Current clamp recordings from dorsal FB neurons in different individuals revealed an unusually wide range of responses to depolarizing current steps: some neurons failed to emit action potentials altogether, even when step-depolarized to positive membrane potentials (Figure 5C); others generated spike trains whose frequency grew in a graded fashion with current amplitude (Figure 5D). The majority of dorsal FB neurons in WT flies belonged to the electrically excitable category (57/80 cells or 71%), whereas the majority of dorsal FB neurons in *cv-c* mutants were electrically silent (25/36 cells or 69%). These figures suggest that Cv-c has a role in setting the intrinsic electrical properties of sleep-promoting neurons.

**Figure 6:**
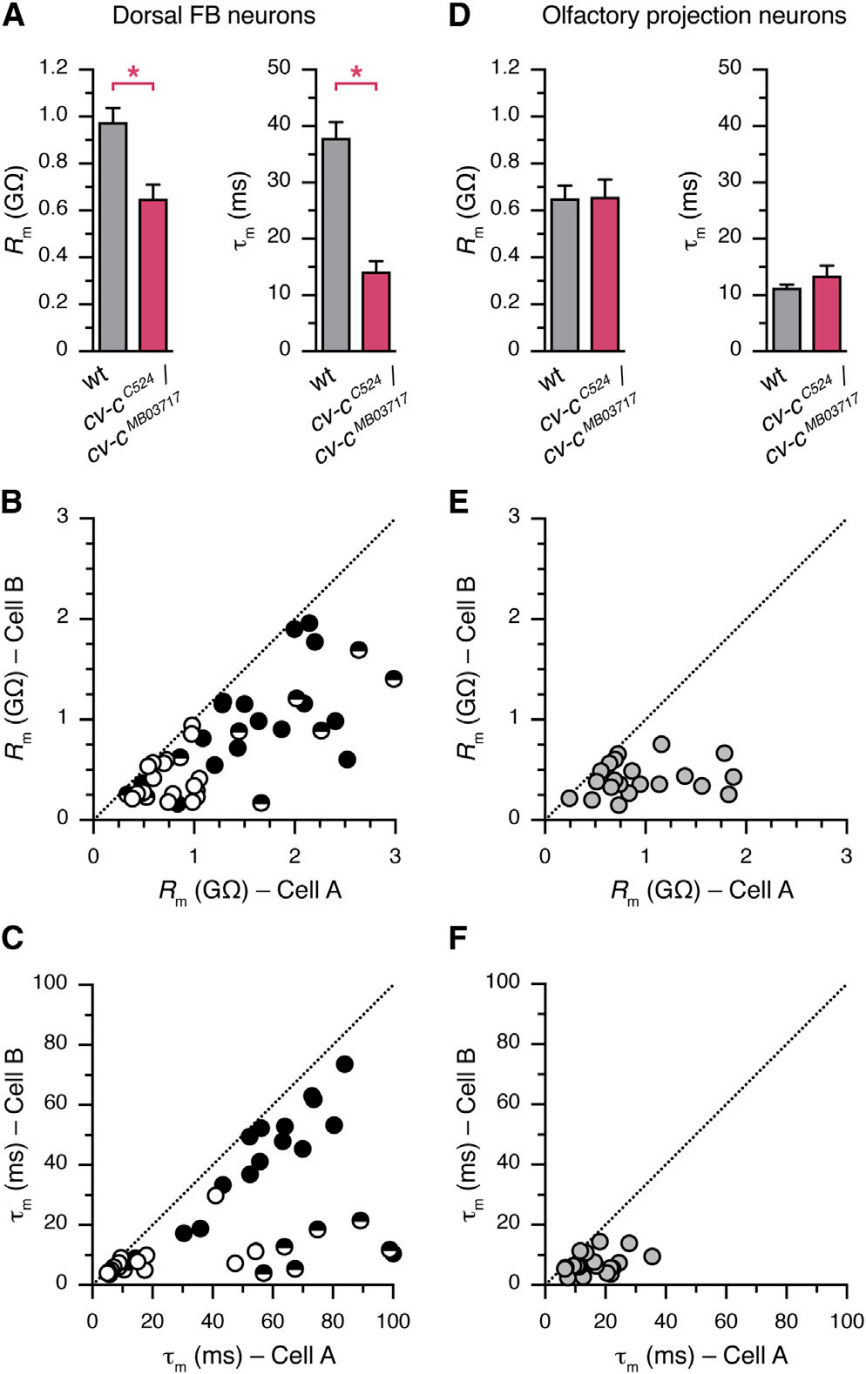
*cv-c* Regulates the Membrane Properties of Sleep-Control Neurons (A) The input resistances (*R*_m_, left) and membrane time constants (τ_m_, right) of dorsal FB neurons differ in WT (gray) and *cv-c*^*C524*^*/cv-c*^*MB03717*^ mutant flies (red; mean ± SEM, n = 37–82 cells per group; two-tailed Student’s t test, p = 0.0026 for *R*_m_ and p < 0.0001 for τ_m_). (B and C) Input resistances (B) and membrane time constants (C) of simultaneously recorded pairs of dorsal FB neurons. The larger value in each pair is by convention assigned to cell A, and all data points therefore lie on or below the dotted identity line. Pairs of two electrically excitable neurons are represented by solid symbols, and pairs of two electrically silent neurons are represented by open symbols. Semifilled symbols denote mixed pairs. Significant correlations exist between members of a pair: R^2^ = 0.5493, p < 0.0001 (*R*_m_) and R^2^ = 0.3306, p < 0.0001 (τ_m_). Note that *R*_m_ and τ_m_ tend to be elevated in electrically excitable neurons, and correlations tend to be weak in mixed pairs. (D) The input resistances (*R*_m_, left) and membrane time constants (τ_m_, right) of olfactory projection neurons do not differ significantly between WT (gray) and *cv-c*^*C524*^*/cv-c*^*MB03717*^ mutant flies (red; mean ± SEM, n = 19–47 cells per group; two-tailed Student’s t test, p = 0.9352 for *R*_m_ and p = 0.3205 for τ_m_). (E and F) Input resistances (E) and membrane time constants (F) of simultaneously recorded pairs of olfactory projection neurons. The larger value in each pair is by convention assigned to cell A, and all data points therefore lie on or below the dotted identity line. There are no significant correlations between members of a pair. R^2^ = 0.0357, p = 0.4119 (*R*_m_) and R^2^ = 0.1100, p = 0.1653 (τ_m_).

Consistent with this idea, dorsal FB neurons in WT and *cv-c* mutant flies differed with respect to two parameters that influence the transformation of synaptic or pacemaker currents into membrane potential changes (Figure 6A). The input resistance, *R*_m_, determines the size of the voltage change caused by the injection of a fixed amount of current (generated, for example, by the activation of synaptic conductances); the membrane time constant, τ_m_, defines the temporal window during which multiple inputs can summate. Both *R*_m_ and τ_m_ were reduced in *cv-c*^*C524*^*/cv-c*^*MB03717*^ mutants in comparison to WT controls (Figure 6A). Thus, the mutation is predicted to decrease the sensitivity of dorsal FB neurons to synaptic inputs and curtail opportunities for input integration over time.

To investigate the extent to which the membrane properties of members of the dorsal FB neuronal population were modulated in concert, we obtained simultaneous recordings from pairs of neurons in the same fly (Figures 6B and 6C). Although spiking and nonspiking neuron types were represented in equal numbers in this data set, the two neurons recorded as part of a pair usually belonged to the same type (36/44 pairs or 81% concordant; χ^2^ = 17.82, 1 degree of freedom, p < 0.0001). This, and significant correlations of input resistance (Figure 6B) and membrane time constant (Figure 6C) between members of a pair, hints that the biophysical properties of different dorsal FB neurons in an individual are coordinated by a common physiological variable, such as the sleep drive of the animal.

Recordings from olfactory projection neurons (PNs) in the antennal lobe suggested that coordinated changes in neuronal membrane properties are neither a common feature of all neurons nor a common side effect of the chronic insomnia of *cv-c* mutants: *R*_m_ and τ_m_ had statistically indistinguishable average values in *cv-c* mutants and WT controls (Figure 6D) and were uncorrelated in pairs of simultaneously recorded PNs (Figures 6E and 6F).

### Sleep Pressure Increases the Excitability of Dorsal FB Neurons

**Figure 7:**
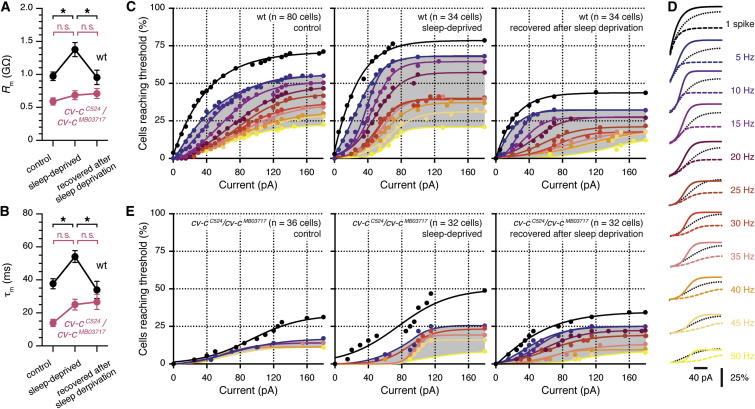
Sleep History Alters the Excitability of Sleep-Control Neurons in a Mechanism Dependent on Cv-c (A) Sleep history alters the input resistance of dorsal FB neurons in WT (black), but not *cv-c*^*C524*^*/cv-c*^*MB03717*^ mutant, flies (red; mean ± SEM, n = 32–81 cells per group). Two-way ANOVA detected significant effects of sleep history (F_[2,243]_ = 4.592, p = 0.0110) and genotype (F_[1,243]_ = 37.93, p < 0.0001) as well as a significant sleep history × genotype interaction (F_[2,243]_ = 3.109, p = 0.0464). Asterisks denote significant differences in pairwise post hoc comparisons. (B) Sleep history alters the membrane time constant of dorsal FB neurons in WT (black), but not *cv-c*^*C524*^*/cv-c*^*MB03717*^ mutant, flies (red; mean ± SEM, n = 29–77 cells per group). Two-way ANOVA detected significant effects of sleep history (F_[2,231]_ = 6.856, p = 0.0013) and genotype (F_[1,231]_ = 38.96, p < 0.0001) as well as a significant sleep history × genotype interaction (F_[2,231]_ = 3.701, p = 0.0262). Asterisks denote significant differences in pairwise post hoc comparisons. (C) Cumulative distribution functions of the percentages of dorsal FB neurons reaching defined spike frequency thresholds (1–50 Hz; see the color code in D) as functions of injected current in WT flies. Left, control flies; center, sleep-deprived flies; right, sleep-deprived flies after 24 hr of recovery sleep. Shaded areas represent the regimes of spike train generation. (D) Superimposition of cumulative distribution functions of the percentages of dorsal FB neurons reaching defined spike frequency thresholds (1–50 Hz) in WT flies. Solid traces that are color coded according to threshold represent the sleep-deprived state, and dashed color-coded traces represent the state after 24 hr of recovery sleep. Dotted black traces indicate flies whose sleep histories have not been experimentally controlled. (E) Cumulative distribution functions of the percentages of dorsal FB neurons reaching defined spike frequency thresholds (1–50 Hz; see the color code in D) as functions of injected current in *cv-c*^*C524*^*/cv-c*^*MB03717*^ mutants. Left, control flies; center, sleep-deprived flies; right, sleep-deprived flies after 24 hr of recovery sleep. Shaded areas represent the regimes of spike train generation. See also Figure S4 and Table S1.

A potential regulatory mechanism thus emerges in which sleep pressure increases the electrical excitability of sleep-promoting neurons in a process that requires the cell-autonomous action of Cv-c. A central prediction of such a mechanism is that rising sleep pressure causes a gain in neuronal responsiveness and that this homeostatic response is blunted in *cv-c* mutants. Indeed, *R*_m_ and τ_m_ of dorsal FB neurons increased in WT flies after overnight sleep deprivation and returned to baseline after sleep-deprived flies had been allowed 24 hr of recovery sleep (Figures 7A and 7B, black). These biophysical changes with immediate sleep history were occluded by *cv-c* ablation; neither *R*_m_ nor τ_m_ varied significantly when short-sleeping *cv-c*^*C524*^*/cv-c*^*MB03717*^ mutants were further sleep deprived or permitted to recover after deprivation (Figures 7A and 7B, red).

To compare patterns of spiking activity between groups of flies, we measured the percentages of cells reaching defined firing rate thresholds during depolarizing current pulses of increasing amplitude (see Figures 5C and 5D for examples). The resulting families of cumulative distribution functions portray the input-output characteristics of dorsal FB neurons in animals with different sleep histories and genetic backgrounds (Figures 7C–7E). In WT flies, sleep deprivation caused a leftward and upward shift of all distribution functions, signaling a broad increase in excitability (Figures 7C and 7D). In comparison to rested animals, identical amounts of current now drove larger percentages of dorsal FB neurons across each spike rate threshold (Figures 7C and 7D; see Table S1 for statistics). This gain in excitability reflects the combined effects of increases in the fraction of neurons that reached each firing rate threshold (cells at plateau, Figure S4A), reductions in the mean current required to recruit one half of the eligible neuronal population at each threshold value (semisaturation current, Figure S4B) and increases in the percentages of cells recruited per current increment (20%–80% slope, Figure S4C).

After a cycle of sleep deprivation that was followed by 24 hr of restorative sleep, the cumulative distribution functions shifted downward and to the right, reflecting a general decrease in excitability (Figures 7C, 7D, and S4). Dorsal FB neurons in flies with experimentally controlled sleep histories thus assumed maxima and minima of electrical responsiveness that may bracket the normal operating range of the population: excitability was maximal immediately after sleep deprivation (Figure 7C, center) and minimal after an extended period of recovery sleep (Figure 7C, right). When neurons were sampled without careful attention to sleep history (Figure 7C, left), their electrical properties tended to fall between these extremes (Figure 7D).

Mutations in *cv-c* not only significantly reduced the spiking activity of dorsal FB neurons in the basal state but also prevented the modulation of excitability after sleep loss: when stepped to depolarized potentials, only ∼20% of all cells in *cv-c*^*C524*^*/cv-c*^*MB03717*^ mutants produced action potential trains (Figure 7E). With the exception of a small rise in the percentage of neurons emitting a single spike, sleep deprivation failed to increase the proportion or responsiveness of electrically excitable cells (Figure 7E). Altogether, these results establish that the baseline activity of dorsal FB neurons and its homeostatic modulation are disrupted in *cv-c* mutants, resulting in deficient sleep.

## Discussion

### The Effector Arm of the Sleep Homeostat

A homeostat (or, in engineering terms, a controller) senses the operation of a system, compares the measured behavior to a set point, computes the necessary corrective action, and actuates the system in order to effect the desired change (Åström and Murray, 2008). The experiments reported here identify sleep-promoting neurons of the dorsal FB as the effector arm of the sleep homeostat. Molecular machinery within these neurons transduces sleep pressure into increased sleep. The output of this machinery, which includes the Rho-GAP encoded by the *cv-c* gene (Figures 3 and 4), is the modulation of membrane excitability (Figures 6 and 7). This modulation appears to be coordinated across the small population of sleep-promoting neurons with axonal projections to the dorsal FB (Figure 6). Spiking of these neurons is, in itself, sufficient for the induction of sleep (Donlea et al., 2011). Given that spike generation is a threshold process, even modest modulation of excitability could result in an effective on/off switch.

Sleep has long been associated with widespread changes in neuronal activity (Brown et al., 2012; Steriade et al., 1993, 2001; Vyazovskiy and Harris, 2013; Vyazovskiy et al., 2009). In sleeping mammals, these changes are reflected in characteristic patterns of extracellular field potentials (Brown et al., 2012; Steriade et al., 1993; Vyazovskiy and Harris, 2013). Several features distinguish these changes from those that are key to the operation of the fly’s sleep homeostat. First, homeostatic sleep control involves a localized increase in the excitability of a circumscribed cluster of sleep-promoting neurons (Figures 6 and 7). This localized gain in electrical responsiveness is in sharp contrast to the diffuse “down” states or “off” periods of reduced activity that commonly accompany non-rapid-eye-movement sleep (Steriade et al., 2001; Vyazovskiy et al., 2009). Second, the homeostatic gain in excitability is a cause and not a consequence of sleep, given that molecular lesions that prevent it lead to insomnia (Figures 1 and 3). No such causal link has been established for any of the other excitability changes that coincide with sleep.

The regulatory logic upstream of the action potential output of the sleep-control neurons is currently unknown. A priori, the remaining elements of the homeostatic feedback loop—sensors, set point, and comparator—could also be housed within the dorsal FB neurons. It is conceivable that these neurons monitor byproducts of prolonged wakefulness, such as changes in the strength of afferent synapses (Tononi and Cirelli, 2003), the release of adenosine (Brown et al., 2012; Porkka-Heiskanen et al., 1997), or rising levels of unfolded proteins or reactive oxygen species (Shaw et al., 2002; Vyazovskiy and Harris, 2013). Once a sensor reports a deviation from the set point that calls for corrective action, the *cv-c*-dependent activity switch is thrown. To do so, the homeostat must compare a physiological variable representing the sleep balance of the organism to a reference representing the homeostatic set point. An obvious cellular mechanism for making such a comparison is the generation of an action potential, which classically involves the application of a fixed criterion to variable synaptic input. Unexpectedly, our recordings demonstrate that it is the ability of sleep-promoting neurons to generate action potentials, and not necessarily the magnitude of the synaptic drive they experience, that varies as a function of sleep need (Figure 7). Given that the excitability of dorsal FB neurons increases during waking, the barrage of synaptic impulses or the force of endogenous pacemaker currents will, at some point, suffice to push the cells across threshold, marking the onset of sleep and thus closing the regulatory loop.

The mechanism we envisage, in which action potential generation by sleep-promoting neurons is a principal variable encoding the sleep balance of the animal, does not exclude that other sleep-related changes take place as well. For example, activity levels in the brain regions providing a synaptic reference signal to the dorsal FB might determine precisely when the transition to sleep occurs, creating a potential link between the intensity of waking experience and the induction of restorative sleep. Several genetic and pharmacological manipulations that target the mushroom bodies delay the onset of sleep, reduce sleep rebound, and enhance performance after extended waking (Donlea et al., 2012; Seugnet et al., 2008, 2011), highlighting one possible source of relevant input signals to the dorsal FB. Arousal-promoting dopaminergic projections to the dorsal FB constitute another (Liu et al., 2012; Ueno et al., 2012). Of course, alternative scenarios are also possible in which sleep need is sensed entirely outside the dorsal FB and communicated to sleep-control neurons via synaptic, metabolic, endocrine, or even glial pathways that feed into cell-intrinsic signaling systems converging on Cv-c.

### The Role of Cv-c in Sleep-Control Neurons

The cell-intrinsic events downstream of Cv-c are already visible in outline. Our measurements of the electrical properties of dorsal FB neurons give a clear indication that sleep history alters the number or conductive properties of ion channels in the plasma membrane of these neurons. When sleep pressure is low, sleep-control neurons have low input resistances and short time constants that are diagnostic of the opening of transmembrane conductances (Figures 5 and 7). These conductances quickly dissipate depolarizing currents, limit the amplitude of membrane potential fluctuations, and oppose spiking. As sleep pressure builds, the gating characteristics of the stabilizing conductances are altered or channels are removed en bloc from the plasma membrane. Input resistances and time constants increase, and excitability also rises.

The loss of functional Cv-c from dorsal FB neurons locks the cells in a high-conductance state that likely corresponds to one extreme of the normal operating range of the sleep homeostat (Figure 7). The inability of mutants to exit this high-conductance state despite intense sleep pressure (Figures 2 and 7) suggests that an essential role of Cv-c is to tune the channel repertoire of sleep-control neurons. Some of the putative substrates of Cv-c, small GTPases of the Rho family (Denholm et al., 2005), have indeed been implicated in various forms of ion channel regulation. RhoA in its active, GTP-bound, membrane-associated state modulates the conductances of delayed rectifier potassium currents (Cachero et al., 1998). Rac1 in its active state promotes the fusion of vesicles containing transient receptor potential channels and thereby increases channel densities in the plasma membrane (Bezzerides et al., 2004). These precedents illustrate the wide range of potential small GTPase substrates, cellular processes, and ion channel targets that future work will have to sift through in order to arrive at a complete molecular description of the sleep homeostat. That said, there still remains a formal possibility that the function of Cv-c in sleep control might be divorced altogether from its catalytic role in the guanine nucleotide cycle of Rho family proteins.

Intriguingly, independent evidence already points to the importance of ion channels in sleep control. Candidate genes identified in mutagenesis or small-molecule screens encode the fast delayed rectifier potassium channel *Shaker* (Cirelli et al., 2005) as well as its cytoplasmic beta subunit *hyperkinetic* (Bushey et al., 2007) and its extracellular regulator *sleepless* (or *quiver*) (Koh et al., 2008), the slow delayed rectifier potassium channel *ether-à-go-go* (Rihel et al., 2010), and the voltage-gated sodium channel *narrow abdomen* (Lear et al., 2005). Our discovery that ion channel modulation in sleep-control neurons lies at the core of sleep homeostasis offers a physiological context for the pursuit of these leads.

## Experimental Procedures

### *Drosophila* Strains and Culture

Fly stocks were grown on standard media of sucrose, yeast, molasses, and agar and maintained on a 12 hr light/12 hr dark schedule. The following strains were used: *cv-c^MB03717^*, *cv-c^MB01956^*, *cv-c^DG20401^* (Bellen et al., 2011; Venken et al., 2011); *cv-c^C524^*, *UAS–cv-c* (Denholm et al., 2005); *UAS–cv-c^RNAi^* (Billuart et al., 2001); *UAS–CD8-GFP* (Lee and Luo, 1999); *C5–GAL4* (Yang et al., 1995); *104y–GAL4* (Rodan et al., 2002; Sakai and Kitamoto, 2006); *C205-GAL4* (Martin et al., 1999); *23E10–GAL4* (Jenett et al., 2012); *tubP–GAL80^ts^* (McGuire et al., 2003).

### Behavior

#### Sleep Measurements

Baseline sleep was measured as previously described (Shaw et al., 2002). In brief, female flies were individually inserted into 65 mm glass tubes, loaded into the TriKinetics *Drosophila* Activity Monitor (DAM) system, and housed under 12 hr light/12 hr dark conditions. Time courses of activity were analyzed by two-way repeated-measures ANOVA; sleep quantities were compared by one-way ANOVA. Arousal thresholds were tested by subjecting flies to mechanical stimuli generated by vibration motors (Precision Microdrives, model 310-113) (van Alphen et al., 2013). Stimuli were delivered for 5 s and separated by 1 hr.

Female flies were sleep deprived by the mechanical SNAP method while housed in TriKinetics DAM systems (Shaw et al., 2002). A cumulative sleep loss plot was calculated for each individual by comparing the percentage of sleep lost during overnight sleep deprivation to the immediately preceding unperturbed night. Individual sleep rebound was quantified hourly for 24 hr by dividing the cumulative amount of sleep regained by the total amount of sleep lost during deprivation. Individual flies were excluded from rebound analysis if sleep deprivation was less than 70% effective or if flies lost less than 60 min of sleep. Statistical significance was assessed by two-way repeated-measures ANOVA.

#### Circadian Analysis

Males were used for circadian analyses in order to prevent interference from developing embryos and larvae over a weeklong experiment. Flies were housed individually in 65 mm glass tubes containing 4% sucrose and 2% agar medium. Locomotor activity was measured in TriKinetics DAM systems for 7 days in constant darkness. χ^2^ periodogram analysis was completed for each individual fly with the ActogramJ plugin (Benjamin Schmid and Taishi Yoshii, University of Würzburg) for ImageJ (National Institutes of Health).

#### Olfactory Memory

Individual female flies were trained and analyzed in 50 mm chambers perfused with air-odor mixtures as previously described (Claridge-Chang et al., 2009). Baseline preference for 3-octanol (OCT) versus 4-methylcyclohexanol (MCH) was measured by tracking a fly’s movements for 2 min. Flies then received two training cycles, which each consisted of two epochs in random order: a 1 min presentation of OCT without shock and a 1 min presentation of MCH with twelve 60-VDC electric shocks. Flies were allowed to recover for 5 min and then reanalyzed for 2 min. Learning is reported as the percentage change in time spent in MCH before and after training (Figure S2).

### Electrophysiology

To gain access for whole-cell patch-clamp recordings in vivo, we removed a small piece of cuticle from the head of female *104y-GAL4*;*UAS-CD8-GFP* flies and targeted the recording electrodes visually to the fluorescent somata of dorsal FB neurons. Control recordings from olfactory PNs were obtained by sampling unlabeled antennal lobe neurons. PNs were distinguished from local neurons by their characteristic electrophysiological properties (Wilson and Laurent, 2005). Borosilicate glass electrodes (7–13 MΩ) were filled with internal solution containing 140 mM potassium aspartate, 10 mM HEPES, 1 mM KCl, 4 mM Mg-ATP, 0.5 mM Na_3_GTP, 1 mM EGTA (pH 7.3), and 0.4% biocytin. The brain was continuously superfused with an extracellular solution containing 103 mM NaCl, 3 mM KCl, 5 mM TES, 8 mM trehalose, 10 mM glucose, 7 mM sucrose, 26 mM NaHCO_3_, 1 mM NaH_2_PO_4_, 1.5 mM CaCl_2_, 4 mM MgCl_2_ (pH 7.3), and continuously equilibrated with 95% O_2_-5% CO_2_. Signals were recorded with a MultiClamp 700B Microelectrode Amplifier, filtered at 6–10 kHz, and digitized at 10–20 kHz with an ITC-18 data acquisition board controlled by the Nclamp and NeuroMatic packages. Data were analyzed with NeuroMatic (http://neuromatic.thinkrandom.com) and custom procedures in Igor Pro (WaveMetrics).

The membrane time constant was determined by fitting a single exponential to the voltage deflection caused by a 200-ms-long hyperpolarizing current pulse. Input resistances were estimated from linear fits of the subthreshold voltage deflections elicited by small current pulses of increasing amplitude and a duration of 1 s. Excitability was quantified by holding cells at resting potentials of –60 ± 2 mV and injecting sequences of depolarizing current pulses (5 pA increments, 1 s duration). Spikes were detected by finding minima in the second derivative of the membrane potential record. The spike rate was calculated by dividing the number of action potentials discharged by the time elapsed between the first and the last spike. Cells that fired only a single action potential per current pulse are denoted as such in Figure 7.

The current amplitude at which each cell reached a given frequency threshold (5–50 Hz) was used to construct cumulative distribution functions. For statistical analyses, the distributions were fit with logistic Naka-Rushton functions (Albrecht and Hamilton, 1982; Naka and Rushton, 1966; Sclar et al., 1990) of the form

$$F=F_{\max}\frac{I^{n}}{I^{n}+I_{50}^{n}},$$

where F is the percentage of cells reaching threshold at a given current level I, $F_{\max}$ is the percentage of cells reaching threshold at maximal current, $I_{50}$ indicates the half-maximal or semisaturation current, and the exponent n determines the steepness of the curve. With only two free parameters ($I_{50}$ and n, given that $F_{\max}$ is measured experimentally), this simple model provided a satisfying fit to all WT distributions (R^2^ > 0.98), irrespective of sleep history. Statistical significance between pairs of distributions was measured with pairwise Kolmogorov-Smirnov (K-S) tests. Because multiple K-S tests were performed, Bonferroni step-down corrections were used. The 20%–80% slope of each cumulative probability distribution was calculated by comparing the 20^th^ and 80^th^ percentiles of the population of cells that reached a particular frequency threshold.

### Confocal Microscopy

For imaging of native GFP fluorescence, brains were dissected in PBS (1.86 mM NaH_2_PO_4_, 8.41 mM Na_2_HPO_4_, and 175 mM NaCl) and fixed for 20 min in 4% paraformaldehyde in PBS at 4°C. Brains containing biocytin fills were incubated in 1:200 streptavidin conjugated to Alexa Fluor 568 (Invitrogen) in PBS containing 0.1% Triton X-100 for 48 hr. Images were collected on a Leica TCS SP5 confocal microscope and processed with ImageJ or Adobe Photoshop.

### Statistics

Statistical analyses were performed with Prism 6 (GraphPad), MATLAB 2009b (MathWorks), or SPSS 22.0.0 (IBM). Pairwise hypotheses were evaluated by Student’s t test. ANOVA, as annotated in Figures 1–7, with Holm-Sidak corrections for multiple comparisons was used in order to test hypotheses involving multiple groups.
