# Neuronal Machinery of Sleep Homeostasis in *Drosophila*

**DOI:** 10.1016/j.neuron.2014.03.008

**Published:** 2014-03-19

**Authors:** Jeffrey M. Donlea, Diogo Pimentel, Gero Miesenböck

(Neuron *81*, 860–872; February 19, 2014)

We thank Douglas Armstrong, Hugo Bellen, Ronald Davis, Ulrike Heberlein, Martin Heisenberg, Liqun Luo, Gerald Rubin, and Helen Skaer for fly strains. In a regrettable oversight, some strains were credited to stock centers rather than the original sources. The correct attributions are as follows:

*cv-c*^*MB03717*^, *cv-c*^*MB01956*^, *cv-c*^*DG20401*^ (Bellen et al., 2011, Venken et al., 2011); *cv-c*^*C524*^, *UAS–cv-c* (Denholm et al., 2005); *UAS–cv-c*^RNAi^ (Billuart et al., 2001); *UAS–CD8-GFP* (Lee and Luo, 1999); *C5–GAL4* (Yang et al., 1995); *104y–GAL4* (Rodan et al., 2002, Sakai and Kitamoto, 2006); *C205-GAL4* (Martin et al., 1999); *23E10–GAL4* (Jenett et al., 2012); *tubP–GAL80*^*ts*^ (McGuire et al., 2003).

The article has been corrected online.
