# Supplementary material for: Neuronal Machinery of Sleep Homeostasis in Drosophila
Source: Neuron. 2014 Feb 19;81(4):860–72. doi: 10.1016/j.neuron.2013.12.013 (PMC3969244; doi:10.1016/j.neuron.2013.12.013)
Supplement: Document S1. Figures S1–S4, and Table S1 [file mmc1.pdf]

Neuron, Volume *81*

Supplemental Information

Neuronal Machinery of Sleep Homeostasis in *Drosophila*

Jeffrey M. Donlea, Diogo Pimentel, and Gero Miesenböck

Supplemental Material

Supplemental Figures and Legends

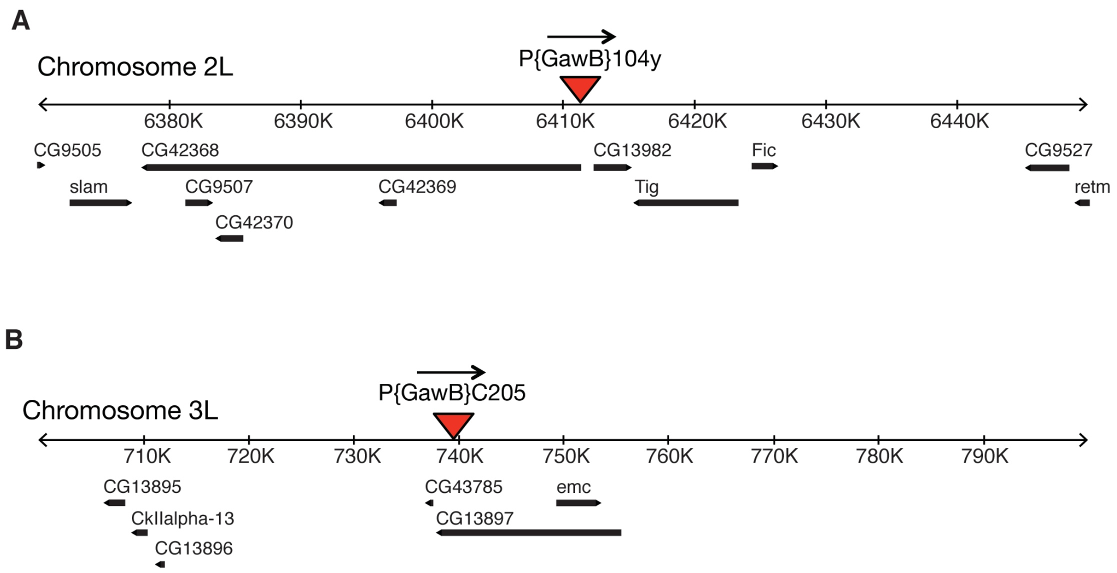

Figure S1, related to Figure 1. Genomic P Element Insertion Sites in *GAL4* Lines Driving Transgene Expression in the Dorsal Fan-Shaped Body.
P element insertion sites (red triangles), as determined by splinkerette PCR, in the *104y-GAL4* (A) and *C205-GAL4* lines (B).

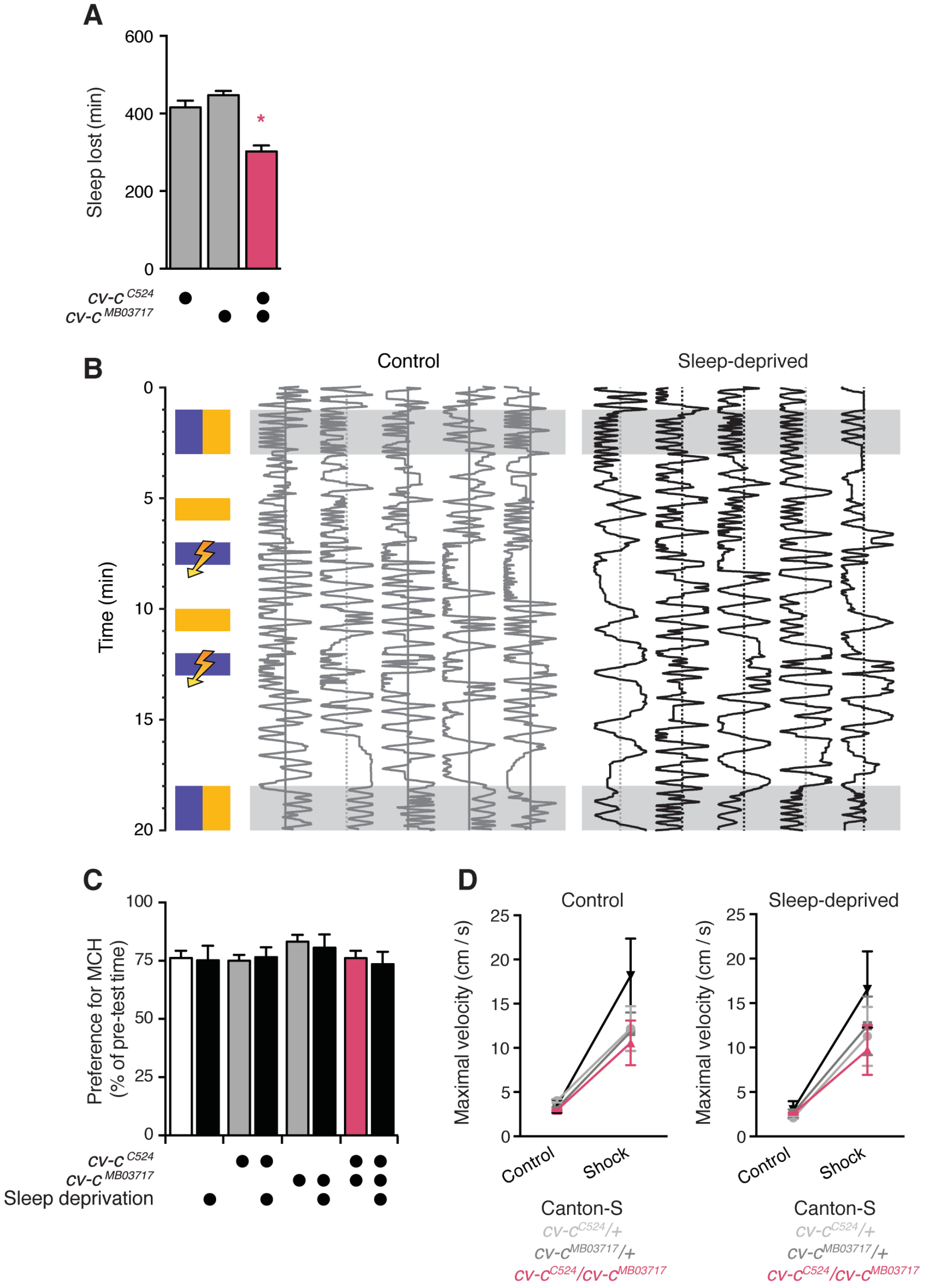

Figure S2, related to Figure 2. Mutations in *cv-c* Impair Homeostatic Sleep Regulation.

(A) Sleep loss of *cv-c*^{C524}/*cv-c*^{MB03717} mutants (red) and heterozygous controls (gray) during 12 h of overnight sleep deprivation. ANOVA detects a significant

genotype effect ($F_{(2, 225)} = 27.67$, $p < 0.0001$). The asterisk denotes a significant difference from heterozygous controls in pairwise post-hoc comparisons.

(B) Example traces of individual flies during a learning and short-term memory experiment. The traces depict the positions of 5 rested (left) and 5 sleep-deprived (right) Canton-S flies in a behavioral chamber (horizontal dimension) as a function of time (vertical dimension). Colored diagrams on the left show epochs of pre- and post-training perference testing between MCH (blue) and OCT (orange). Training cycles consist of two 1 min presentations of the individual odors. Pairing the presentations of MCH with electric shock causes conditioned avoidance of MCH in the rested, but not in the sleep-deprived, flies shown.

(C) Percentage of time spent in MCH before training. Two-way ANOVA detects no significant effects of genotype ($F_{(3, 328)} = 1.36$, $p = 0.2566$) or sleep history ($F_{(1, 328)} = 0.16$, $p = 0.6900$).

(D) Maximal motor velocities of rested (left) or sleep-deprived (right) Canton-S flies (black), $cv\text{-}c^{C524}/cv\text{-}c^{MB03717}$ mutants (red), and heterozygous controls (gray) before and during exposure to electric shock. Three-way ANOVA detects a significant effect of electric shock ($F_{(1, 648)} = 78.26$, $p < 0.0001$) but not of sleep history ($F_{(1, 648)} = 0.42$, $p = 0.5165$) or genotype ($F_{(3, 648)} = 2.00$, $p = 0.1128$).

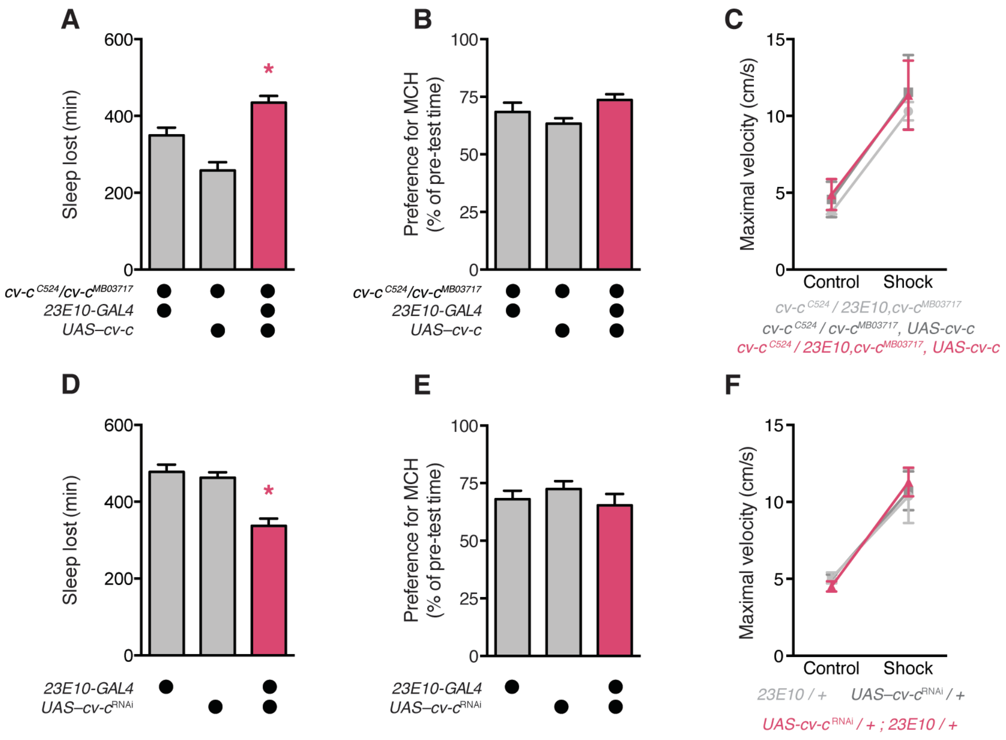

Figure S3, related to Figure 4. Deficits in Homeostatic Sleep Control and Memory Reflect the Role of Cv-c in the Dorsal Fan-Shaped Body

(A) Sleep loss of *cv-c*^{C524}/*23E10,cv-c*^{MB03717},*UAS-cv-c* rescue flies (red) and parental controls (gray) during 12 h of overnight sleep deprivation. ANOVA detects a significant genotype effect ($F_{(2,169)} = 20.08$, $p < 0.0001$). The asterisk denotes a significant difference from both parental controls in pairwise post-hoc comparisons.

(B) Percentage of time spent in MCH before training. ANOVA detects a significant genotype effect ($F_{(2,192)} = 4.43$, $p = 0.0131$), due to a difference between *cv-c*^{C524}/*23E10,cv-c*^{MB03717},*UAS-cv-c* rescue flies and one set of parental controls (*cv-c*^{C524}/*cv-c*^{MB03717},*UAS-cv-c*).

(C) Maximal motor velocities of *cv-c*^{C524}/*23E10,cv-c*^{MB03717},*UAS-cv-c* rescue flies (red) and parental controls (gray) before and during exposure to electric shock. Repeated measures ANOVA detects a significant effect of electric shock ($F_{(1,192)} = 21.41$, $p < 0.0001$) but not of genotype ($F_{(2,192)} = 0.23$, $p = 0.7952$).

(D) Sleep loss of *UAS-cv-c*^{RNAi}/+ ; *23E10*/+ experimental flies (red) and parental controls (gray) during 12 h of overnight sleep deprivation. ANOVA detects a

significant genotype effect ($F_{(2,189)}$ = 16.67, p < 0.0001). The asterisk denotes a significant difference from both parental controls in pairwise post-hoc comparisons.

(E) Percentage of time spent in MCH before training. ANOVA fails to detect a significant genotype effect ($F_{(2,117)}$ = 0.7693, p = 0.4657).

(F) Maximal motor velocities of *UAS-cv-c*^{RNAi}/+ ; *23E10*/+ experimental flies (red) and parental controls (gray) before and during exposure to electric shock. Repeated measures ANOVA detects a significant effect of electric shock ($F_{(1,117)}$ = 58.56, p < 0.0001) but not of genotype ($F_{(2,117)}$ = 0.01629, p = 0.9838).

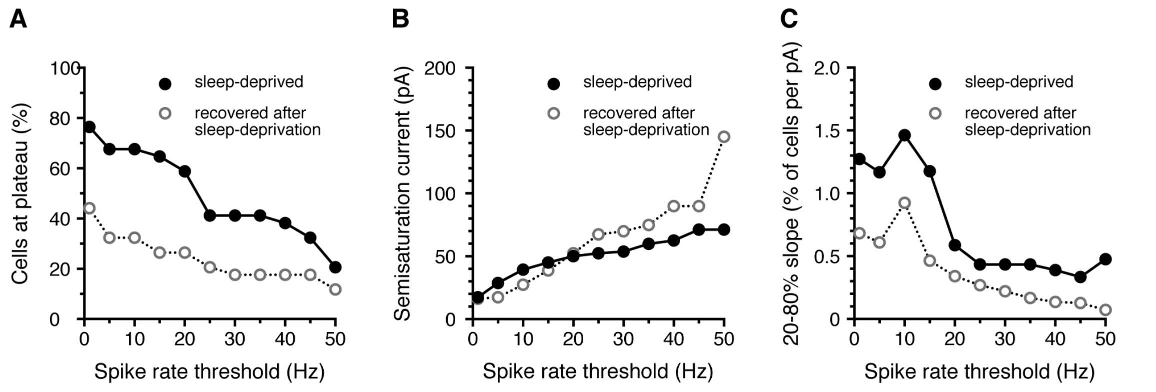

Figure S4, related to Figure 7. Parameters of Spike Train Generation.
(A–C) Percentages of neurons at plateau (A), semisaturation currents (B), and 20-80% slopes (C) as functions of spike rate thresholds in wild-type flies. Solid and open symbols represent, respectively, conditions after sleep deprivation and 24 h of recovery sleep.

Supplemental Table

Table S1, related to Figure 7. Pairwise Comparisons of Cumulative Frequency Distributions of Spike Rates by Kolmogorov-Smirnov (K-S) Test.

Frequency threshold	Control vs. sleep-deprived	Sleep-deprived vs. recovered	Control vs. recovered
1 Hz	K-S D = 0.6905<br>$p < 0.0001$	K-S D = 0.8333<br>$p < 0.0001$	K-S D = 0.8095<br>$p < 0.0001$
5 Hz	K-S D = 0.7381<br>$p < 0.0001$	K-S D = 0.8571<br>$p < 0.0001$	K-S D = 0.7857<br>$p < 0.0001$
10 Hz	K-S D = 0.7381<br>$p < 0.0001$	K-S D = 0.8095<br>$p < 0.0001$	K-S D = 0.7381<br>$p < 0.0001$
15 Hz	K-S D = 0.6905<br>$p < 0.0001$	K-S D = 0.7857<br>$p < 0.0001$	K-S D = 0.6667<br>$p < 0.0001$
20 Hz	K-S D = 0.5476<br>$p < 0.0001$	K-S D = 0.7619<br>$p < 0.0001$	K-S D = 0.5714<br>$p < 0.0001$
25 Hz	K-S D = 0.3333<br>$p = 0.0188$	K-S D = 0.7381<br>$p < 0.0001$	K-S D = 0.5714<br>$p < 0.0001$
30 Hz	K-S D = 0.4762<br>$p = 0.0001$	K-S D = 0.7381<br>$p < 0.0001$	K-S D = 0.6190<br>$p < 0.0001$
35 Hz	K-S D = 0.4762<br>$p = 0.0001$	K-S D = 0.7143<br>$p < 0.0001$	K-S D = 0.5952<br>$p < 0.0001$
40 Hz	K-S D = 0.5238<br>$p < 0.0001$	K-S D = 0.6905<br>$p < 0.0001$	K-S D = 0.5714<br>$p < 0.0001$
45 Hz	K-S D = 0.3333<br>$p = 0.0188$	K-S D = 0.6429<br>$p < 0.0001$	K-S D = 0.5238<br>$p < 0.0001$
50 Hz	K-S D = 0.3095<br>$p = 0.0358$	K-S D = 0.6429<br>$p < 0.0001$	K-S D = 0.5952<br>$p < 0.0001$
